# Effect of percutaneous nucleoplasty with coblation on phospholipase A2 activity in the intervertebral disks of an animal model of intervertebral disk degeneration: a randomized controlled trial

**DOI:** 10.1186/s13018-015-0175-y

**Published:** 2015-03-25

**Authors:** Dajiang Ren, Zhicheng Zhang, Tiansheng Sun, Fang Li

**Affiliations:** Department of Orthopedic Surgery, Beijing Army General Hospital, Nanmencang No. 5, Dongcheng District, Beijing, 100700 China

**Keywords:** Intervertebral disk degeneration, Low back pain, Coblation, Nucleoplasty, Animal model, Phospholipase A2

## Abstract

**Background:**

This randomized controlled trial was carried out to (1) evaluate the effect of nucleoplasty with coblation on the PLA2 activity in the degenerative intervertebral disks of an animal model and (2) explore the possible therapeutic mechanism of coblation in addition to the current theory, which focuses on decreasing the intradiskal pressure in the treatment of intervertebral disk degeneration.

**Methods:**

Thirty-six animal models of intervertebral disk degeneration were successfully established and then randomly divided into two groups: the coblation group (*n* = 18) and coblation control group (*n* = 18). Nucleoplasty using coblation was performed in the coblation group. L4–5 and L5–6 intervertebral disk samples were harvested and analyzed for PLA2 activity in different groups at different time points.

**Results:**

The PLA2 activity in the coblation control group was significantly higher than that in the control group (194.86 ± 11.80 and 80.68 ± 5.56, respectively; *P* < 0.01). There was a significant decrease in the PLA2 activity 1 week after coblation than at the real time after coblation (154.39 ± 7.99 and 184.98 ± 9.43, respectively; *P* < 0.001). The PLA2 activity at 1 month after coblation remained at a lower level than those at 1 week and at the real time after coblation (142.63 ± 10.72, 154.39 ± 7.99, and 184.98 ± 9.43, respectively), but there was no significant decrease in the PLA2 activity between 1 week and 1 month after coblation.

**Conclusions:**

Coblation appeared to effectively degrade the PLA2 activity in the degenerative intervertebral disks of this animal model. This represents a potential mechanism for the clinical use of coblation in the treatment of low back pain.

## Introduction

Low back pain remains a major threat to public health worldwide. It is generally caused by degeneration of the intervertebral disks. The typical mechanism of pain, described as compression of the nerve root by the herniated disk, reportedly cannot explain all of the clinical symptoms. The concept of local chemical mediation of pain by the injured tissues has recently gained much more favor. Many cytokines that may be responsible for chemical mediation of low back pain have been identified [1-3]. Findings in animal studies and clinical research suggest that phospholipase A2 (PLA2) is not only an important inflammatory cytokine and pain-causing factor but also a special signature of local tissue inflammation. PLA2 activity is closely associated with intervertebral disk degeneration, intervertebral disk herniation, radicular pain, and lumbar diskogenic pain. It is the initial factor in a series of pathophysiological changes and has an acknowledged proinflammatory effect [3-6]. Therefore, PLA2 plays an important role in the pain associated with disk degeneration.

Less invasive techniques introduced in recent years, including percutaneous nucleoplasty, are based on high-tech equipment and medications and contribute to less pain and more effective clinical outcomes in the treatment of low back pain. These minimally invasive techniques have been gradually accepted by both surgeons and patients [7]. The therapeutic mechanism of percutaneous nucleoplasty is thought to ablate the nucleus pulposus and decrease the intradiskal pressure. Since 2002, we have used this technique to treat more than 800 patients with low back pain. We found that some patients with low back pain without high intradiskal pressure (such as severe intervertebral disk degeneration or annulus fibrosus tears) could also be cured or obtain satisfactory pain relief after coblation therapy. The therapeutic mechanism of coblation in these patients was unclear. Therefore, this study was designed to explore the effect of coblation on the PLA2 activity in degenerative intervertebral disks and provide experimental evidence for the clinical use of coblation in the treatment of low back pain due to intervertebral disk degeneration.

## Methods

### Experimental animals

**Figure 1:**
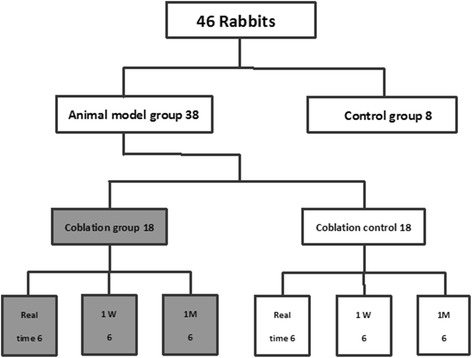
**Animal groups.** Two rabbits died of infection during establishment of the animal model. Thirty-six animal models were successfully established. All animal models were then randomly divided into a coblation group and coblation control group. Six animals were sacrificed at different time points to assess the PLA2 activity in the L4–5 and L5–6 intervertebral disks.

This study was approved by the Animal Experimental Ethics Committee of Beijing Army General Hospital. A total of 46 New Zealand white rabbits (weight, 2.0–2.5 kg; age, 2.0–2.5 months) were purchased from Vital River Laboratories (Beijing, China). They were randomly assigned to an animal model group (*n* = 38) and a control group (*n* = 8). A surgical procedure was performed in the animal model group to establish the animal model of intervertebral disk degeneration. Two rabbits died of infection after the surgery. Thirty-six animal models were successfully established and then randomly divided into two groups: the coblation group (*n* = 18) and coblation control group (*n* = 18). At each time point, six rabbits in the coblation and coblation control groups were euthanized for the analysis of PLA2 activity (Figure 1). Food and drinking water were available *ad libitum*.

### Establishment of the animal model

All rabbits were anesthetized with sodium pentobarbital (35 mg/kg, intraperitoneal injection). Magnetic resonance imaging (MRI) (Signa TwinSpeed 1.5 T Excite II; GE Healthcare, USA) and X-ray were performed to confirm the preoperative status of the involved disks prior to surgery. The animal model of intervertebral disk degeneration was induced as previously described [8]. A C-arm with image intensification (BV300; Philips Electronics, Eindhoven, The Netherlands) was used to identify the location of L4–5 and L5–6. A posterolateral incision was made in the iliac crest, and the L4–5 and L5–6 disks were exposed. A 16-gauge puncture needle was used to induce an injury to the disk to a depth of 5 mm. Muscles and skin were closed with 3-0 silk suture. Four weeks postoperatively, the animal model group underwent MRI and X-ray examinations to assess the degeneration of the disks, and 36 animal models were successfully established. Two animals died of iatrogenic infection. The MR images of the disk was assessed by an observer blinded to the study using modified Thompson classification based on changes in the degree and area of signal intensity from grades 1 to 4 (1 = normal, 2 = minimal decrease in signal intensity but obvious narrowing of high-signal, 3 = moderate decrease in signal intensity, 4 = severe decrease in signal intensity). The animal model with grade 2 or grade 3 was suitable for the study.

### Nucleoplasty procedure

Nucleoplasty (Coblation®; ArthroCare Spine, USA) was performed in the coblation group as soon as we identified the successful establishment of the animal model of intervertebral disk degeneration, while no procedure was performed in the coblation control group. Each rabbit was tranquilized as described above. The operative field was prepared in a sterile fashion using betadine. L4–5 and L5–6 were identified with the C-arm while using Kirschner wires as markers. The procedure was performed as follows. The skin was stabbed by a 16-gauge hypodermic needle in preparation for puncture. Under fluoroscopic guidance, the access needle was inserted at an angle of 40° from the mid-back, targeting the tip of the stylet to the center of the nucleus in both the coronal and sagittal planes. The exact positioning of the stylet was then confirmed using anteroposterior and lateral views. The stylet was withdrawn from the needle, and a DC SpineWand was inserted under fluoroscopic guidance. The SpineWand was then connected to the cable. The controller was set at power level 1. The COAG pedal on the foot controller was depressed for 0.5 s. The ABLATION pedal on the foot controller was depressed for 3 to 5 s while the flange was rotated 180° in a back-and-forth motion. At the same time, the effective length was confirmed to extend from the inner part of the annulus fibrosus to that of the opposite side. The depth of penetration was controlled to extend not more than 5 mm from the needle tip. After the coblation zone had been created, the SpineWand was withdrawn from the needle and the needle was then withdrawn from the rabbit. Throughout all procedures, care was taken to avoid disturbing the peripheral tissues around the vertebrae. Following the surgeries, the rabbits were permitted free cage activity (4,000 cm^2^), food, and water. Gentamicin was given intramuscularly for three consecutive days.

### Assessment of PLA2 activity in the intervertebral disk

The PLA2 activity assessment (titrimetric analysis) was performed as previously published [9]. All reagents were purchased from Beijing Chemical Preparation Company (Beijing, China). The substrate buffer solution comprised phosphatidylcholine (3.75 mmol/L), glycine (0.1 mmol/L), boric acid (3.57 mmol/L), and sodium deoxycholate (6.03 mmol/L). Before use, the solution was placed in a water bath at 60°C for 30 min, and the pH was adjusted to 8.50 with sodium hydroxide solution (1 mol/L). The components of the PLA2 diluent were similar to those of the substrate buffer solution, but phosphatidylcholine was not included. Dilute hydrochloric acid (0.005 mmol/L) was also prepared for titration and determination of the amount of base.

The rabbits in the coblation and coblation control groups were followed serially by both MRI and X-ray at different time points: real time, 1 week, and 4 weeks after nucleoplasty. At each of these time points, six rabbits were randomly euthanized for analysis of disk specimens. A posterior longitudinal skin incision was made along the lumbar spine in a sterile fashion. Both the L4–5 and L5–6 motion segments were dissected from the lumbar spine. The L4–5 and L5–6 intervertebral disks were then carefully separated with a scalpel. Each disk was cut into 1-mm^3^ pieces. After being weighed, the disk tissue was mixed with PLA2 diluent (4 ml/g) and centrifuged at 4,000 r/min for 10 min. The supernatant was mixed with 0.4 ml of PLA2 diluent, centrifuged at 8,000 r/min for 20 min, and collected and stored at 4°C.

First, each sample was placed in a 60°C thermostatic water bath for 30 min. Next, 0.4-ml samples and different reagents according to Table 1 were added to two tubes (tubes A and B), then placed in a water bath at 37°C for 60 min. Tube A was added with 0.2 ml calcium chloride (0.2 ml), and ethylenediaminetetraacetic acid (EDTA) (1.1 ml) was added to tube B. The pH in tube A was carefully adjusted to match that in tube B by titration with dilute hydrochloric acid. A sensitivity pH meter (sensitivity, 0.001) (inoLab pH 740; WTW Co., Weilheim, Germany) was used to detect the endpoint of the reaction. The PLA2 activity was considered to indicate the consumption of dilute hydrochloric acid.

**Table 1:** **Procedure of PLA2 activity measurement**

**Regents**	**Tube A**	**Tube B**
Substrate buffer solution	8 ml	8 ml
Calcium chloride (0.5 mol/L)	-	0.2 ml
EDTA^a^ (15 mmol/L)	1.1 ml	-
Samples	0.4 ml	0.4 ml
	Water bath at 37°C, 60 min	
Calcium chloride (0.5 mol/L)	0.2 ml	-
EDTA (15 mmol/L)	-	1.1 ml

One PLA2 activity unit was defined as follows: 1 ml of sample consumes 1 nmol of dilute hydrochloric acid solution per min during titration at 37°C. (1 U = l nmol ml/min). ^a^Ethylenediaminetetraacetic acid.

$$ \mathrm{P}\mathrm{L}\mathrm{A}2(U)=\frac{N\kern0.5em \times \kern0.5em V\kern0.5em \times \kern0.5em {10}^6\kern0.5em \times \kern0.5em 2.5}{t} $$

(*V*: volume of hydrochloric acid solution consumed; *N*: concentration of hydrochloric acid solution; *t*: time of titration).

### Statistical analysis

All data were statistically analyzed with SPSS 16.0 (SPSS, Inc., Chicago, IL, USA). The data are presented as mean ± standard deviation (SD). The PLA2 activity between the coblation control group and control group was compared using Student’s *t*-test. One-way analysis of variance or Fisher’s least significant difference test was used to compare the differences in the PLA2 activities at different time points in the coblation group. A *P* value of <0.05 was considered statistically significant.

## Results

### MRI and plain radiography of animal model of intervertebral disk degeneration

**Figure 2:**
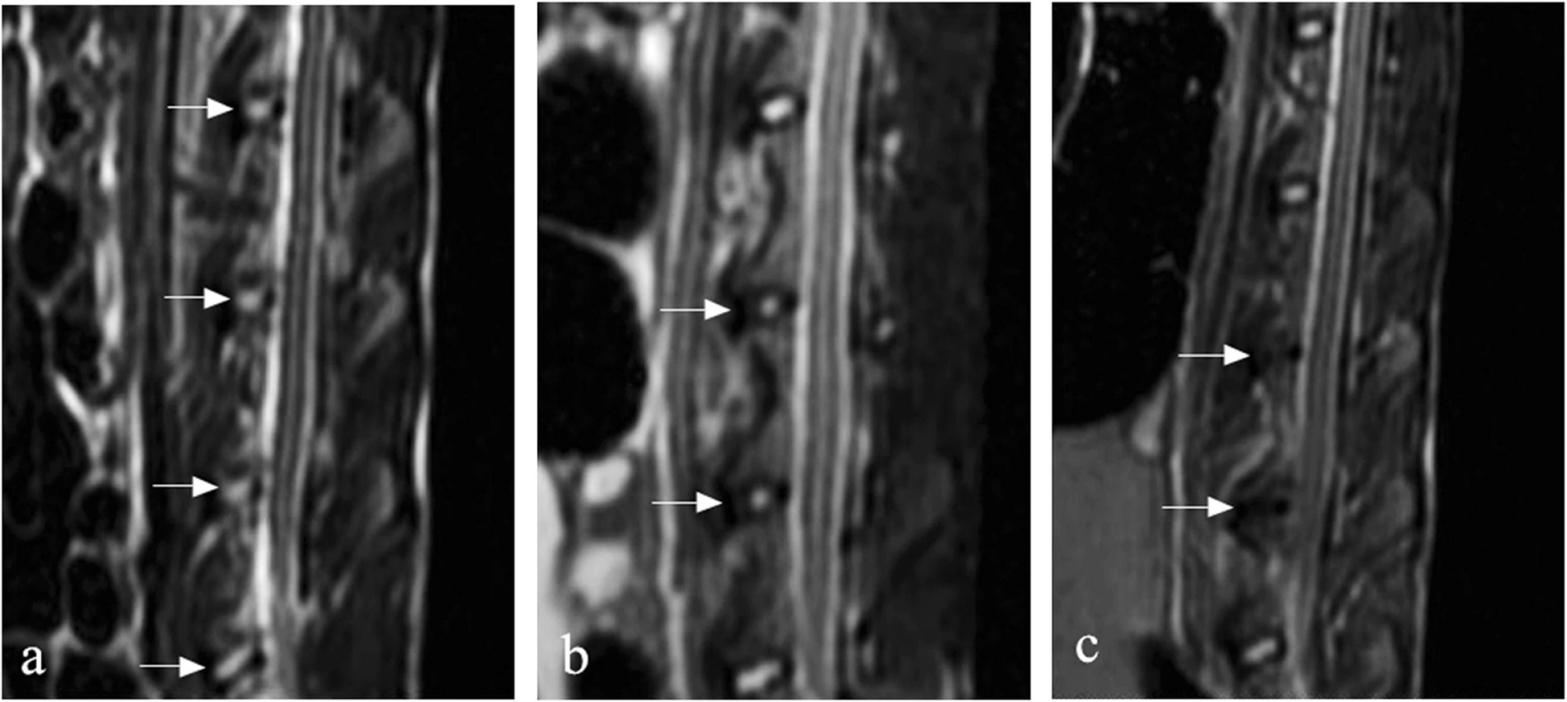
**Signal intensity of the disks in animal model group. (a)** T2-weighted spin-echo images show four normal intervertebral disk signals in the control group (white arrows). **(b)** Two degenerative intervertebral disks in the coblation group with lower signal intensity than normal disks (1 month after stabbing; original magnification × 2). **(c)** T2-weighted spin-echo images show two “black disks” with disk intensity of grade 4 in the coblation control group at 8 weeks after stabbing (white arrows, original magnification × 1.5).

Degenerative changes in L4–5 and L5–6 were identified in 36 rabbits by MRI and plain radiography compared with the control group. All the signal intensity of the disks in animal model group was between grade 2 and grade 3 at 4 weeks after stabbing. Severe degeneration could be observed in the coblation control group at 8 weeks after stabbing (Figure 2).

**Figure 3:**
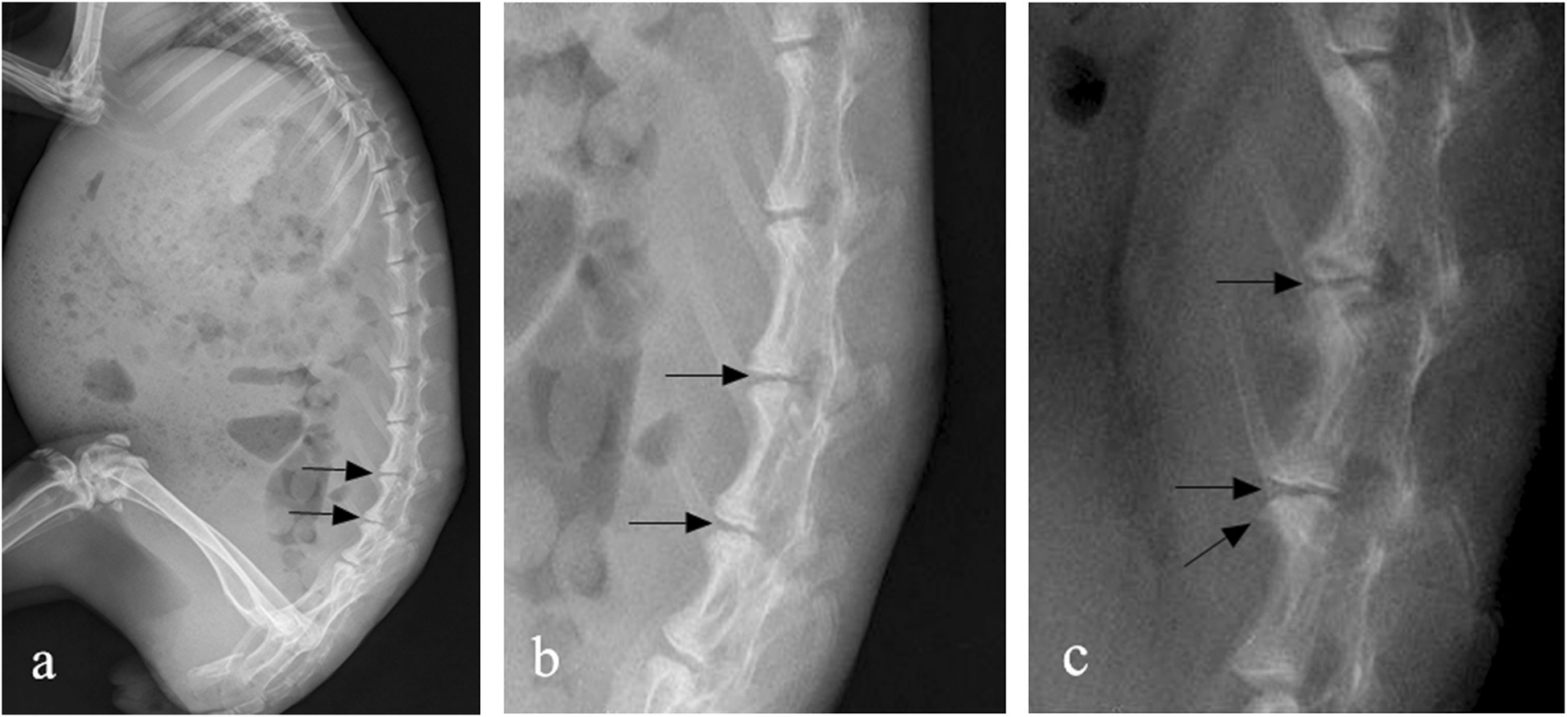
**Lateral radiographs showing L4–5 and L5–6. (a)** Reduced disk height of L4–5 and L5–6 on lateral plain radiographs at 4 weeks after stabbing (black arrows). **(b)** Neither osteophyte formation nor endplate thickening were observed during this period, only reduced disk height is apparent (original magnification × 2.5). **(c)** Further loss in disk height was seen at 8 weeks after stabbing (black arrows). Early osteophyte formation (oblique arrow) was observed in this period, but endplate thickening still did not appear (original magnification × 2.0).

Reduced disk height of L4–5 and L5–6 and early osteophyte formation were shown on lateral radiographs. Endplate thickening (sclerosis) was not observed at any time point (Figure 3).

### Effect of coblation on PLA2 activity in intervertebral disks

The PLA2 activity in the intervertebral disks of different groups is shown in Table 2. The PLA2 activity in the coblation control group was significantly higher than that in the control group (194.86 ± 11.80 vs. 80.68 ± 5.56, respectively; *P* < 0.01). When coblation was performed on the degenerative intervertebral disks, the PLA2 activity in the coblation group was significantly lower than that in the coblation control group (184.98 ± 9.43 vs. 194.86 ± 11.80, respectively; *P* < 0.05). In the coblation group, the PLA2 activity developed a gradually declining trend (real time, 184.98 ± 9.43; >1 week, 154.39 ± 7.99; >1 month, 142.63 ± 10.72), but a significant difference in PLA2 activity was only noted between real time and 1 week after coblation (184.98 ± 9.43 vs. 154.39 ± 7.99, respectively; *P* < 0.001).

**Table 2:** **PLA2 activity in intervertebral disks at different time points among different groups**

**Groups**	**PLA2 activity (U)**
Control group	80.68 ± 5.56
Coblation group	
Real time	184.98 ± 9.43
1 week later	154.39 ± 7.99
1 month later	142.63 ± 10.72
Coblation control group	
Real time	194.86 ± 11.80
1 week later	195.20 ± 13.90
1 month later	225.98 ± 12.59

## Discussion

Intervertebral disk degeneration is a multifaceted, chronic process involving certain detrimental, progressive changes in disk composition, structure, and function that occur more quickly and/or with greater severity than those associated with normal aging [10]. It has been widely studied with respect to its potential involvement in the onset of low back pain. The mechanical behaviors of intervertebral disk degeneration and their changes with degeneration have been investigated, but the current mechanical theory cannot explain all treatment effects. For instance, the pathophysiology of sciatica due to lumbar disk herniation involves factors other than mere mechanical compression of the nerve root because many patients with disk herniation remain entirely asymptomatic. The pain-related chemical mediators released by the tissues involved in degenerative disk disease have been increasingly recognized in recent years [11,12]. Many inflammatory cytokines are related to disk degeneration. These factors appear to be activated in cases of symptomatic herniation, suggesting that inflammation of the nerve root might result from an inflammatory process in the herniated disk itself. Among them, PLA2, a lipolytic enzyme first discovered in the herniated disk tissues in 1990 by Saal et al. [13], preferentially cleaves arachidonic acid from membrane phospholipids, resulting in various inflammatory mediators such as prostaglandins and leukotrienes. It has played an important role as a very important inflammatory cytokine within the pathological manifestations of the disk degeneration, neurological symptoms, peripheral nerve injury, and diskogenic pain [14-17].

In our study, we found that the PLA2 activity in the coblation control group was significantly higher than that in the control group, indicating that PLA2 activity is closely related to intervertebral disk degeneration. One postulated mechanism is that when intervertebral disk degeneration occurs, PLA2 is activated by various proinflammatory mediators such as interleukin-1, tumor necrosis factor-α, and interleukin-6, which are secreted by the degenerative intervertebral disk. This gradually leads to high expression of PLA2 in the degenerative intervertebral disk as the degeneration progresses.

Over recent years, several minimally invasive treatment modalities for intervertebral disk degenerative disease have been studied, such as intradiskal steroid injection, intradiskal electrothermal therapy, and intradiskal radiofrequency thermocoagulation. Coblation is a surgical method of volumetric tissue removal through molecular dissociation during arthroscopic surgery developed by ArthroCare Corporation. It achieves its operative result through a lower intradiskal pressure. In practical applications, however, we have found that the current theory of mechanical decompression cannot explain all clinical phenomena. In some patients with low back pain, the contrast media (injected volume of >2 ml) spread rapidly outside the annulus fibrosus during diskography, indicating severe intervertebral disk degeneration or an annulus fibrosus tear. Interestingly, these patients also achieved satisfactory pain relief after coblation therapy. Under this premise, we postulated that pain relief is associated with decreased inflammatory cytokine levels after coblation.

The PLA2 activity in the coblation control group was significantly higher than that in the control group. When coblation was performed on degenerative intervertebral disks, the PLA2 activity began to decline over time. In addition, a significant difference in PLA2 activity in the coblation group was only noted between real time and 1 week after coblation. Accordingly, it can be postulated that coblation energy has an effect on the PLA2 activity in degenerative intervertebral disks, especially in the early treatment stage.

Based on the results obtained in this study, we conclude that the energy produced by coblation can degrade the PLA2 activity in the intervertebral disk, resulting in pain relief in the treatment of low back pain, although the exact mechanism remains unclear. A possible explanation is that the energized particles during the coblation procedure have sufficient energy to break down the molecular bonds of PLA2. As a result, the PLA2 activity decreased after the treatment. Because PLA2 is considered to be the rate-limiting enzyme in the inflammatory cascade reaction, the progressive inflammatory effect was terminated by the destruction of PLA2 activity. Notably, the PLA2 activity obviously decreased 1 week after the procedure and gradually declined over time. Therefore, it can be postulated that inhibition of the inflammatory cascade reaction is an important mechanism of treatment by way of terminating PLA2 activity.

There are several limitations in this study. (1) The observation period was 4 weeks long. Further research with longer study periods should be conducted to identify changes in PLA2 activity. (2) Histological analysis was not performed to examine the morphological features of the cells in the intervertebral disk in this study; such features could be related to changes in PLA2 activity. (3) PLA2 activity was detected by titrimetric analysis instead of immunohistochemical analysis, which could have led to bias with respect to methodology.

In conclusion, PLA2 activity is closely associated with the different stages of intervertebral disk degeneration. According to this study, we found that low PLA2 expression is present in normal intervertebral disks, and PLA2 activity gradually increases with further progression of degeneration. Percutaneous nucleoplasty is a relatively novel therapeutic method that was introduced in recent years. In our experimental study, percutaneous nucleoplasty was performed based on the successful establishment of an animal model of intervertebral disk degeneration. We have found that coblation can degrade the PLA2 activity in degenerative intervertebral disks. The therapeutic effect appears to be significant 1 week postoperatively then slowly declines over time. The therapeutic effects of coblation in patients with severe degenerative intervertebral disks and annulus fibrosus tears can be explained through this experimental study using an animal model.
